# Collecting Duct-Specific CR6-Interacting Factor-1-Deletion Aggravates Renal Inflammation and Fibrosis Induced by Unilateral Ureteral Obstruction

**DOI:** 10.3390/ijms222111699

**Published:** 2021-10-28

**Authors:** Jin Young Jeong, Ki Ryang Na, Jin Ah Shin, Kwang-Sun Suh, Jwa-Jin Kim, Kang Wook Lee, Dae Eun Choi

**Affiliations:** 1 Department of Nephrology, Chungnam National University School of Medicine, Daejeon 35015, Korea; spwlsdud@naver.com (J.Y.J.); drngr@cnu.ac.kr (K.R.N.); kjj4827@gmail.com (J.-J.K.); kwlee@cnu.ac.kr (K.W.L.); 2 Department of Medical Science, Chungnam National University School of Medicine, Daejeon 35015, Korea; wlsdkahh@gmail.com; 3 Department of Pathology, Chungnam National University School of Medicine, Daejeon 35015, Korea; kssuh@cnu.ac.kr

**Keywords:** CRIF1, mitochondria, Cre-loxp, collecting duct, kidney, inflammation, fibrosis

## Abstract

Although inflammation and fibrosis, which are key mechanisms of chronic kidney disease, are associated with mitochondrial damage, little is known about the effects of mitochondrial damage on the collecting duct in renal inflammation and fibrosis. To generate collecting duct-specific mitochondrial injury mouse models, CR6-interacting factor-1 (CRIF1) ^flox/flox^ mice were bred with Hoxb7-Cre mice. We evaluated the phenotype of these mice. To evaluate the effects on unilateral ureteral obstruction (UUO)-induced renal injury, we divided the mice into the following four groups: a CRIF1^flox/flox^ (wild-type (WT)) group, a CRIF1^flox/flox^-Hob7 Cre (CRIF1-KO) group, a WT-UUO group, and a CRIF1-KO UUO group. We evaluated the blood and urine chemistries, inflammatory and fibrosis markers, light microscopy, and electron microscopy of the kidneys. The inhibition of *Crif1* mRNA in mIMCD cells reduced oxygen consumption and membrane potential. No significant differences in blood and urine chemistries were observed between WT and CRIF1-KO mice. In UUO mice, monocyte chemoattractant protein-1 and osteopontin expression, number of F4/80 positive cells, transforming growth factor-β and α-smooth muscle actin staining, and Masson’s trichrome staining were significantly higher in the kidneys of CRIF1-KO mice compared with the kidneys of WT mice. In sham mice, urinary 8-hydroxydeoxyguanosine (8-OHDG) was higher in CRIF1-KO mice than in WT mice. Moreover, CRIF1-KO sham mice had increased 8-OHDG-positive cell recruitment compared with WT-sham mice. CRIF1-KO-UUO kidneys had increased recruitment of 8-OHDG-positive cells compared with WT-UUO kidneys. In conclusion, collecting duct-specific mitochondrial injury increased oxidative stress. Oxidative stress associated with mitochondrial damage may aggravate UUO-induced renal injury.

## 1. Introduction

Renal inflammation and fibrosis are common final pathways in chronic kidney disease (CKD) [1]. In CKD, the proximal tubules are vulnerable to damage, leading to inflammation and fibrosis caused by ischemia, toxic substances, and oxidative stress [2]. The contribution of the collecting duct to renal inflammation and fibrosis is relatively unknown. Although damage to the collecting duct caused by a specific genetic modification causes kidney fibrosis [3,4], the role of mitochondrial damage in the collecting duct is unknown in various CKD models.

Mitochondria play a central role in cellular respiration, regulation of reactive oxygen species (ROS), and signal transduction of apoptosis [5]. Although various tubule interstitial injury models, including models of ischemia-reperfusion renal injury, cisplatin nephrotoxicity, and diabetic nephropathy, have shown functional or structural mitochondrial abnormalities, few studies have focused on whether mitochondrial injury itself aggravates the injuries [6,7,8]. Furthermore, the role of mitochondria in kidney injury is not confirmed. One study has shown that inhibitors of the respiratory complex system in mitochondria via hypoxia aggravated cisplatin-induced kidney injury [9]. However, another study has shown a contradictory report that inhibition of the mitochondrial respiratory complex system via hypoxia attenuated cisplatin-induced kidney injury [10].

Although CR6-interacting factor 1 (CRIF1) is a nuclear protein that regulates the cell cycle by binding to the Gadd45 family [11,12], our previous study showed that CRIF1 deficiency leads to mitochondrial dysfunction both functionally and structurally [13]. We induced collecting duct cell-specific CRIF1 deletion using the Cre-loxP system with HoxB7 as a promoter and developed mouse models with mitochondrial structural abnormalities limited to collecting tubular epithelial cells. We evaluated functional and structural abnormalities in kidneys in mouse models of collecting duct-specific mitochondrial injury. Moreover, using a unilateral ureteral obstruction (UUO) model, we attempted to determine the effects of collecting duct-specific-mitochondrial injury on renal tubular interstitial inflammation and fibrosis.

## 2. Results

### 2.1. CRIF1-KO Mice Showed Extensive Mitochondrial Destruction of Collecting Duct Cells

**Figure 1:**
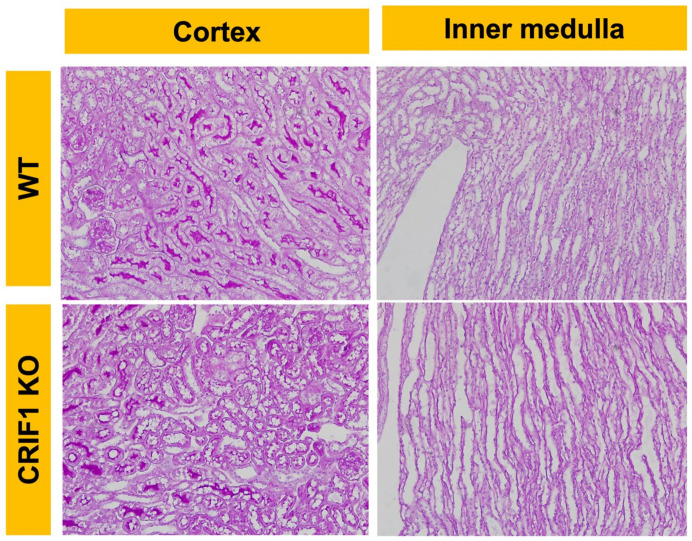
Photos of periodic acid–Schiff stain of wild-type (WT) and CR6-interacting factor-1 knockout (CRIF1-KO) mouse kidneys. No specific morphological differences are observed between WT and CRIF1-KO mice.

**Figure 2:**
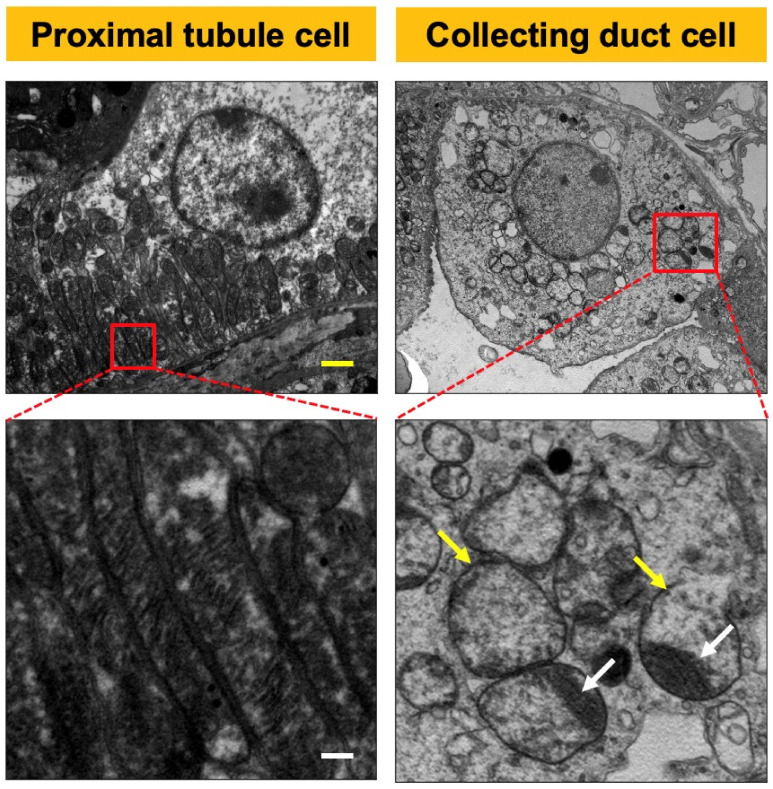
Photos of electron microscopy of CR6-interacting factor-1 knockout (CRIF1-KO) sham mouse kidney. The mitochondria of collecting duct cells were swollen (white arrow) and showed the destruction of cristae (yellow arrow) in the kidneys of CRIF1-KO mice. The mitochondria of proximal tubular cells showed normal morphology in CRIF1-KO mice. Yellow scale bar, 2 μm. White scale bar, 0.2 μm.

On light microscopy, no specific morphological differences were observed between wild-type (WT) mice and the CRIF1flox^/flox^ Hoxb7-Cre genotype (CRIF1-KO) mice in 10-week-old male mice (Figure 1). On electron microscopy, the mitochondria of 10-week-old male CRIF1-KO mice showed extensive destruction of cristae structure, were swollen, and had more collecting duct cells. Other tubules, including the proximal and distal tubules, had normal mitochondria (Figure 2).

### 2.2. Silencing RNA of CRIF1-Induced Mitochondrial Dysfunction in Inner Medullary Collecting Duct (mIMCD) Cells

**Figure 3:**
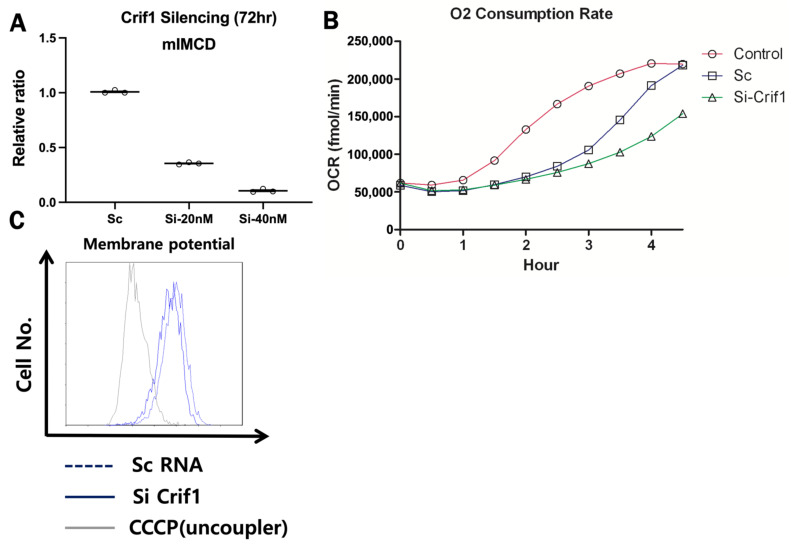
Representative mRNA expression of CR6-interacting factor-1 (CRIF1) in mIMCD cells (**A**). CRIF1 mRNA expression is decreased with silencing RNA treatment. O2 consumption and membrane potential are significantly decreased in Si-CRIF1-treated mIMCD cells compared with sc-treated and control mIMCD cells (**B**,**C**).

*Crif1* RNA silencing effectively reduced mRNA expression of *Crif1* in mIMCD cells. Silencing *Crif1* RNA significantly reduced mitochondrial membrane potential and O2 consumption rate compared with scramble RNA treatment (Figure 3).

### 2.3. Phenotype of CRIF1-KO Mice

**Figure 4:**
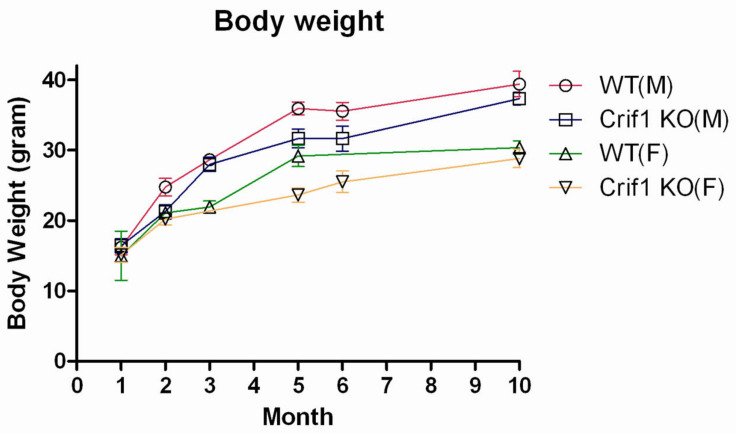
Body weight of wild-type (WT) and CR6-interacting factor-1 knockout (CRIF1-KO) mice.

No significant differences in body weight, arterial pH, BUN, s-Cr, s-Na, s-K, s-Cl, urine urea nitrogen, u-Cr, u-Na, u-K, and u-Cl were observed between CRIF1-KO and WT mice in 10-week-old mice (Figure 4 and Table 1). Additionally, there were no significant differences in body weight and urine output between CRIF1-KO and WT mice in the 6th month and 10th month. 

### 2.4. Effects of Mitochondrial Dysfunction of Collecting Duct Cells on UUO-Induced Renal Injury

**Figure 5:**
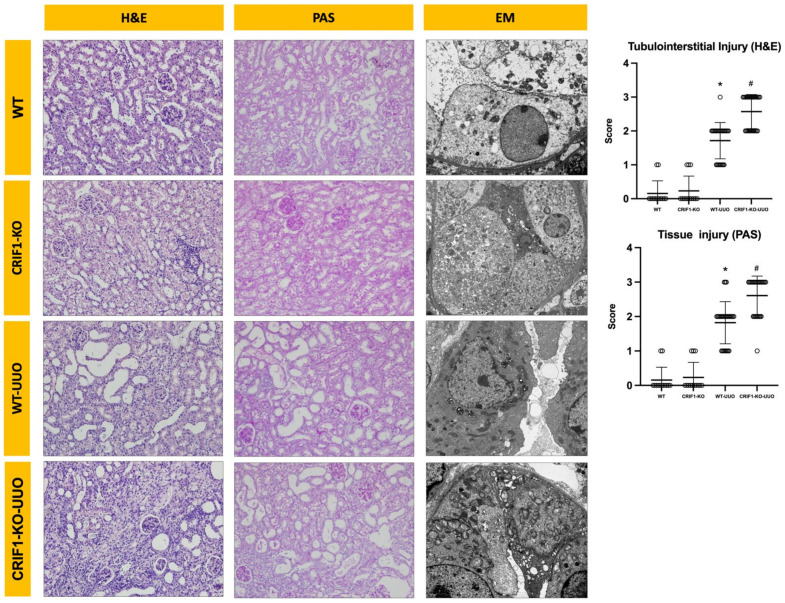
Photos of hematoxylin and eosin, periodic acid–Schiff stains, and electron microscopy of wild-type (WT) and CR6-interacting factor-1 knockout (CRIF1-KO) mouse kidneys. WT-unilateral ureteral obstruction (UUO) kidneys showed an increase in tubular injury score compared with non-UUO kidneys. CRIF1-KO-UUO kidneys showed an increase in tubular injury score compared with WT-UUO kidneys. Although CRIF1-KO kidneys showed severe mitochondrial injury, UUO did not significantly alter the mitochondrial morphology in WT kidneys. * *p* < 0.05, WT-sham vs. WT-UUO; # *p* < 0.05, WT-UUO vs. CRIF1-KO-UUO.

The infiltration of monocytes in the interstitium, tubules, and desquamation of epithelial cells in the renal tubular epithelium were significantly increased in WT-UUO mice compared with those in control mice. These injuries were significantly aggravated in the kidneys of CRIF1-KO-UUO mice (Figure 5).

**Figure 6:**
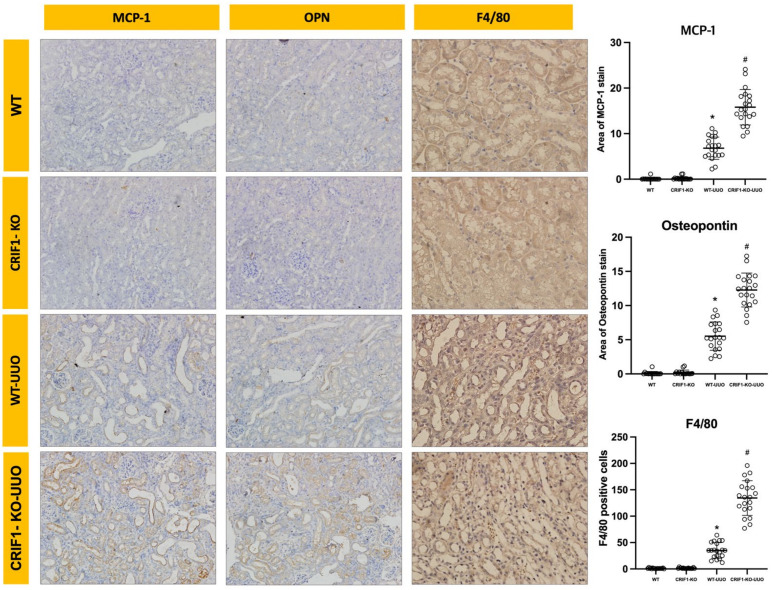
Photos of immunohistochemical stain of MCP-1, osteopontin (OPN), and F4/80. CR6-interacting factor-1 knockout (CRIF1-KO)-unilateral ureteral obstruction (UUO) kidneys showed an increase in the stained areas of MCP-1 and OPN and the number of F4/80-positive cells compared with wild-type (WT)-UUO kidneys. * *p* < 0.05, WT-sham vs. WT-UUO; # *p* < 0.05, WT-UUO vs. CRIF1-KO-UUO.

For evaluating inflammation, we examined OPN and MCP-1 expression, which are typical macrophage chemokines. OPN and MCP staining showed significantly broader areas in the kidneys of CRIF1-KO-UUO mice than in those of WT-UUO mice. Furthermore, we examined F4/80 expression using immunohistochemistry to evaluate macrophage infiltration. Immunostaining for F4/80 showed that CRIF1-KO-UUO mice had significantly increased macrophage (F4/80-positive cells) recruitment compared with WT-UUO mice; however, no significant differences in F4/80 immunostaining were observed between CRIF1-KO and WT mice (Figure 6, Supplement Figure S1).

**Figure 7:**
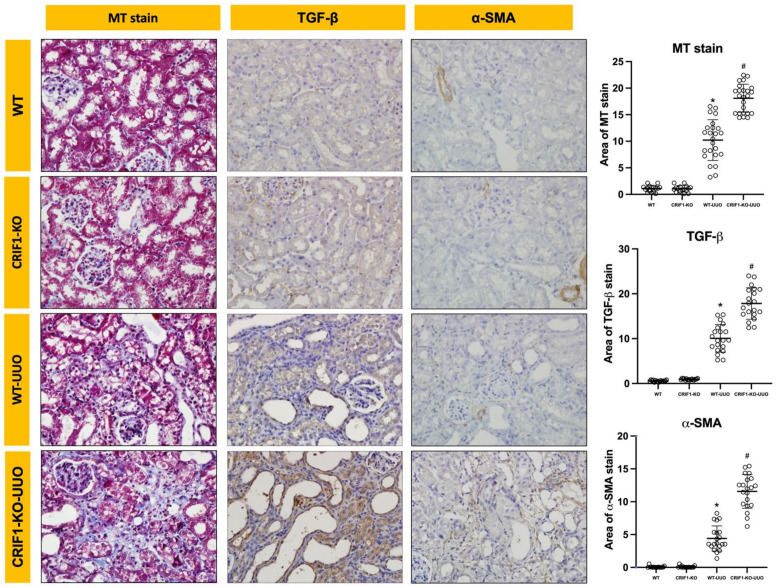
Photos of Masson’s trichrome and immunohistochemical stains of TGF-β and α-SMA. CR6-interacting factor-1 knockout (CRIF1-KO)-unilateral ureteral obstruction (UUO) kidneys showed an increase in the stained areas of TGF-β and α-SMA and Masson’s trichrome-stained area compared with wild-type (WT) UUO kidneys. * *p* < 0.05, WT-sham vs. WT-UUO; # *p* < 0.05, WT-UUO vs. CRIF1-KO-UUO.

To evaluate fibrosis, we evaluated TGF-β, α-SMA, and Masson’s trichrome staining. TGF-β- and α-SMA- staining showed significantly broader areas in the kidneys of CRIF1-KO-UUO mice than in WT-UUO mice. In Masson’s trichrome staining, CRIF1-KO-UUO kidneys showed more extensive fibrosis than WT-UUO kidneys (Figure 7, Supplement Figure S2).

### 2.5. The Effect of Oxidative Stress on UUO-Induced Injury

**Figure 8:**
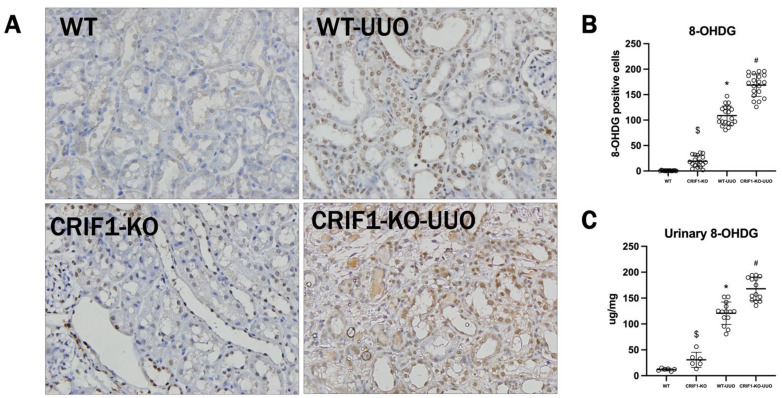
Photos of immunohistochemistry of 8-hydroxydeoxyguanosine (8-OHDG) (**A**). CR6-interacting factor-1 knockout (CRIF1-KO) mice had a significant increase in 8-OHDG-positive cell recruitment compared with wild-type (WT) mice. CRIF1-KO-unilateral ureteral obstruction (UUO) kidneys showed higher recruitment of 8-OHDG-positive cells than WT-UUO kidneys (**B**). Urinary 8-OHDG excretion in WT and CRIF1-KO mice (**C**). CRIF1-KO mice had a significant increase in urinary 8-OHDG compared with WT mice. CRIF1-KO-UUO kidneys showed higher urinary 8-OHDG than WT-UUO kidneys. $ *p* < 0.05, WT-sham vs. CRIF1-KO-sham; * *p* < 0.05, WT-sham vs. WT-UUO; # *p* < 0.05, WT-UUO vs. CRIF1-KO-UUO.

In immunostaining of 8-hydroxydeoxyguanosine (8-OHDG) in the kidneys, CRIF1-KO mice had a significantly increased 8-OHDG-positive cell recruitment compared with WT mice, and UUO-induced renal injury increased 8-OHDG-positive cell recruitment compared with that in WT mice. Moreover, CRIF1-KO-UUO kidneys had increased recruitment of 8-OHDG-positive cells compared with WT-UUO kidneys. Urinary 8-OHDG was significantly increased in CRIF1-KO mice compared with that in WT mice. UUO mice showed significantly increased urinary 8-OHDG compared with sham mice. Furthermore, CRIF1-KO-UUO mice showed a significantly increased urinary 8-OHDG compared with WT-UUO mice (Figure 8).

## 3. Discussion

Here, we developed collecting duct-specific mitochondrial injury models and their phenotypes. Furthermore, we showed the effects of mitochondrial injury to collecting duct cells on tubulin interstitial fibrosis induced by UUO.

Collecting ducts contribute to the regulation of acid-base balance, the concentration of urine, and the handling of Na, K, and Cl [14]. Furthermore, some active processes, including ion transport, need adenosine triphosphate, which is mainly produced in the mitochondria. However, in this study, collecting duct-specific mitochondrial injury did not affect ion and water transport, including urine volume, blood and urine chemistries, expression of ion transport, and pH of blood gas, except for the decrease in urinary pH. Although the mitochondria are important energy sources for collecting duct cells, blood supply coming from arteries, including the arcuate artery and pars recta, may supply sufficient energy [15].

To evaluate the role of mitochondrial dysfunction in tubule interstitial injury, we used UUO models. UUO is a well-established model of experimental renal injury that results in inflammation and fibrosis of the tubulointerstitial area showing macrophage infiltration and fibroblast activation or proliferation [16]. Furthermore, oxidative stress contributes to renal tubulointerstitial inflammation and epithelial-to-mesenchymal transition and fibrosis in UUO models [17,18].

In renal injury models, various ROS sources are implicated, including the mitochondrial respiratory chain [19,20], NADPH oxidase [21], xanthine oxidase, cyclooxygenase, lipoxygenase [22], and uncoupled nitric oxide synthase [23]. Despite the appreciable evidence of oxidant stress involvement in UUO, few have been investigated for each oxidative stress source in renal injury. Because oxidative stress mechanisms vary between models, identifying specific ROS sources that may be potential treatment targets is important. Mitochondria are major sources of intracellular ROS, and oxidative damage to cardiolipin in the inner mitochondrial membrane promotes mitochondrial permeability transition and cytochrome *c* release, resulting in cellular damage [24,25,26]. In this study, baseline oxidative stress was increased in CRIF1-KO sham mice compared with that in WT-sham mice. Furthermore, oxidative stress was higher in the kidneys of CRIF1-KO UUO mice than in the kidneys of WT-UUO mice. Although CRIF1-KO sham mice showed collecting duct mitochondrial destruction and increased 8-OHDG in the urine and kidney tissue compared with WT-sham mice, no significant differences in inflammatory and fibrosis markers and cellular morphology in hematoxylin and eosin (H&E) and periodic acid–Schiff (PAS) staining were observed between WT and CRIF1-KO sham mice. Furthermore, the kidneys of CRIF1-KO UUO mice showed a larger extent of loss of brush border tubular cell detachment and tubular cell atrophy than those of WT-UUO mice. 

Although oxidative stress plays a key role in various kidney injuries, including ischemic reperfusion injury, diabetic kidney injury, and chronic kidney injury, its role in kidney damage when oxidative stress increases due to mitochondrial damage alone under normal conditions is not well known [27]. In our previous study, podocyte-specific mitochondrial damage induced massive proteinuria and glomerulosclerosis [13]. However, collecting duct-specific mitochondrial damage did not show significant abnormalities in renal function and cellular structure in this study. These results suggest the possibility that oxidative stress in the collecting duct alone does not increase renal inflammation and fibrosis. In a mitochondrial injury environment, when fibrotic factors, such as UUO, are added, it causes greater damage, suggesting that mitochondrial dysfunction in the collecting duct makes the tubular cells more susceptible to UUO-induced cellular damage and death, resulting in the aggravation of inflammatory cell infiltration and progression of renal fibrosis.

Taken together, *Crif1* deletion induced the destruction of the mitochondria structurally and functionally. Collecting duct-specific mitochondrial injury aggravates tubulointerstitial inflammation and fibrosis in UUO kidneys.

## 4. Materials and Methods

### 4.1. Animals and UUO Operation

All experiments were performed on C57BL/6 background mice weighing 28–30 g. We obtained the HoxB7 Cre mice and *Crif1* flox mice from Dr. Kong and Dr. Shong, respectively. A standard laboratory diet and water were provided, and the mice were cared for according to the protocol approved by the Institutional Animal Care and Use Committee of the Chungnam National University (IRB NO.CNU-00455). For evaluation of baseline parameters, the urine of 10-week-old mice (WT mice, n = 7, and CIRF1 KO mice, n = 8) was collected for 24 h using a metabolic cage. The blood of 10-week-old male mice, from 0.9 to 1.1ml was collected through the cardiac LV puncture under anesthesia for arterial blood gas analysis and biochemical tests. The mice were anesthetized using intraperitoneal ketamine (2 mg/kg, Ketalar; Bayer, Leverkusen, Germany) and xylazine (200 µL/kg; Rompun; Bayer). We evaluated the mice’s body weight.

We divided the 10-week-old male mice into the following four groups: CRIF1flox/flox (WT) group (n = 7); CRIF1 flox/flox-Hob7 Cre (CRIF1-KO) group (n = 7); WT-UUO group (n = 8); and CRIF1-KO UUO group (n = 8). Under anesthesia, the left ureter was exposed via a lateral incision and ligated using two sutures at the level of the lower renal pole for UUO operation. Urine was collected from the mice through metabolic cages on the 6th and 7th days after the operation. The mouse kidneys were obtained through an abdominal incision under anesthesia, then mice were euthanized.

### 4.2. Development of CRIF1-KO Mouse Models

**Figure 9:**
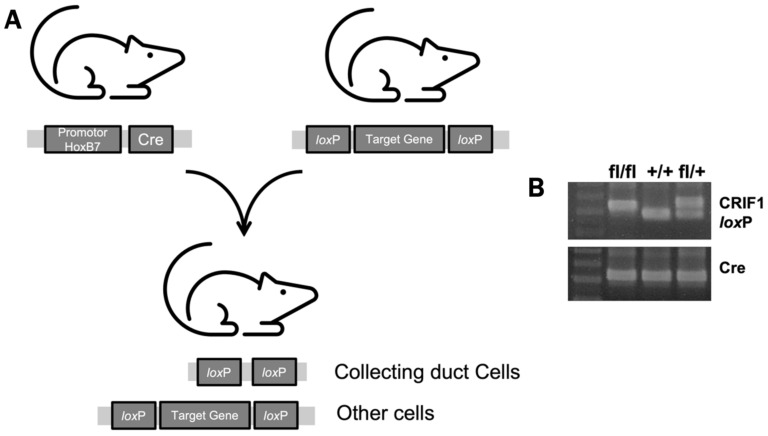
Generation of CR6-interacting factor-1 knockout (CRIF1-KO) mice. After CRIF1 flox/flox mice bred with HoxB7 mice (**A**), the genotype of siblings was evaluated using polymerase chain reaction (**B**).

CRIF1 flox/flox mice were bred with Hoxb7-Cre mice, which express Cre recombinase exclusively in the collecting duct epithelium of the kidneys and the epithelium of small parts of the dorsal root ganglia and spinal cord. The CRIF1flox/flox Hoxb7-Cre genotype was confirmed using polymerase chain reaction (PCR) (Figure 9).

### 4.3. Measurement of Blood and Urine Chemistries

To evaluate blood and urine chemistries, we examined urea nitrogen, creatinine, sodium, potassium, chloride, and serum and urine osmolarity using the chemistry autoanalyzer Toshiba 200FR (Toshiba Medical Systems Co., Tokyo, Japan). Furthermore, we evaluated arterial blood gases using a blood gas analyzer (GEM premier 3000 model 5700; Instrumentation Laboratory, San Diego, CA, USA). Then, we evaluated the urine pH (Seven Easy pH, Mettler Toledo, Greifensee, Switzerland).

### 4.4. Measurement of Urine 8-OHDG

Urine levels of 8-OHDG, an oxidized nucleoside of DNA, were determined using a commercial enzyme-linked immunosorbent assay (ELISA) kit (Japan Institute for the Control of Aging, Nikken SEIL Co., Ltd., Shizuoka, Japan) according to the manufacturer’s instructions. Absorbance at 450 nm was measured using an ELISA reader (Molecular Devices, Inc., San Jose, CA, USA).

### 4.5. Tissue Preparation

At the end of the study, the mice were anesthetized, and the kidneys were perfused briefly through the abdominal aorta with cold saline to rinse the blood. The left kidney was immediately excised. A piece of the kidney was fixed in 10% buffered formaldehyde at room temperature and then embedded in Paraplast (Sherwood Medical, St. Louis, MO, USA) for light microscopy and immunohistochemistry.

### 4.6. Light Microscopy Examination

Pieces of the embedded kidney were cut into 4 μm sections and mounted onto glass slides. The sections were deparaffinized with xylene, stained with H&E, PAS, and Masson’s trichrome, and examined under an Olympus BX51 microscope (Olympus, Tokyo, Japan). The kidney tissue injury score ranged from 0 to 3 and was categorized as follows: 0, normal kidney; 1, mild changes; 2, moderate changes; and 3, severe changes. Ten fields of the outer medulla were evaluated. The renal fibrotic areas were quantified by morphometric analysis using a light microscope and *digital camera-based image analyzer* (Image-Pro plus 5.3, Mediacybernetics, Bethesda, MD, USA). Blue-positive areas (fibrotic areas) were quantified and evaluated by computer-based morphometric analysis.

### 4.7. Electron Microscopic Examination

Fresh tissue samples were processed immediately and fixed with 2.5% glutaraldehyde for more than 8 h and cleaned using a buffer solution. Then, they were fixed with osmic acid and dehydrated, embedded, and polymerized. The ultra-thin sections were made and dyed for transmission electron microscopic observation (Transmission Electron Microscope, HITACHI, Tokyo, Japan).

### 4.8. Cell Culture

mIMCD, an immortalized collecting duct cell line, was purchased from the American Type Culture Collection (Manassas, VA, USA) and cultured. mIMCD-3 cells were grown in Dulbecco’s modified Eagle’s medium/Ham’s F-12(1:1) (Lonza, Walkersville, MD, USA), supplemented with 10% fetal bovine serum (Lonza, Walkersville, MD, USA), 100 U/mL penicillin (Lonza, Walkersville, MD, USA), and 100 μg/mL streptomycin (Lonza, Walkersville, MD, USA) at 37 °C in a humidified atmosphere of 95% air and 5% CO_2_.

### 4.9. Small Interfering RNA (siRNA) Experiments

Stealth siRNA duplex oligonucleotides were synthesized by Invitrogen. Stealth RNAi Negative Control (Medium GC, Invitrogen) was used as a non-silencing control. Before transfecting siRNA, cells were plated at 70% confluency in Dulbecco’s modified Eagle’s medium supplemented with 10% fetal bovine serum, 100 U/mL penicillin, and 100 μg/mL streptomycin and incubated overnight. Transfections were performed using HiPerFect Transfection Reagent (QIAGEN, Hilden, Germany) as directed by the manufacturer.

### 4.10. RNA Extraction, Semi-Quantitative Reverse Transcription PCR, and Real-Time PCR

Total RNA was extracted from cultured cells using a Nucleospin RNA II kit (Macherey–Nagel, Düren, Germany). The cDNA was synthesized from 2.5 μg total RNA using an oligo dT primer (ELPIS, Daejeon, Korea), dNTPs (ELPIS), M-MLV reverse transcriptase (Invitrogen), 0.1 M DTT, and buffers in a total reaction volume of 20 μL. Twenty microliters of cDNA was diluted to a total volume of 100 μL. PCR was used to amplify the following specific cDNAs: CRIF1 (sense: 5′-TAT CTC CTG CGG CTC TCT GT-3′; antisense: 5′-CTT CTG CTT TCG CCA GTT TT-3′). PCR was performed using SYBR Green PCR Master Mix (QIAGEN). The amplification reaction volume was 20 μL, which consisted of 10 μL iQ SYBR Green PCR MasterMix, 2 μL primers, 2 μL cDNA, and 6 μL H**_2_**O. Amplification and detection were performed using a thermal cycler (Rotor-Gene 6000; Corbett Research, Mortlake, Australia). PCR conditions were as follows: denaturation at 95 °C for 10 min and 40 amplification cycles of 10 s at 95 °C, 15 s at 55 °C, and 20 s at 72 °C. The fluorescence of SYBR Green was measured at the end of each cycle using the comparative threshold cycle (Ct) method. The quantification of cDNA was determined using the following arithmetic formula for each group of data: 2^−ΔΔCt^ = 2 − ((Ct of target gene-Ct of GAPDH in control, scRNA-treated, and siRNA-treated mIMDC cells) − (Ct of target gene − Ct of GAPDH in control mIMCD cells)).

### 4.11. Mitochondrial Function Test

*O2 consumption:* Whole cells were harvested and washed with PBS. The whole-cell pellets were then suspended in phosphate-buffered saline at a concentration of 2 × 10^6^ cells/mL. Then, 200 μL of each cell suspension was placed in individual wells of a BD Oxygen Biosensor System plate (BD Biosciences, San Jose, CA, USA). Each suspension was run in triplicate. The BD Oxygen Biosensor System plates contain a dye that fluoresces with exposure to dissolved oxygen. The plates were scanned using a Fluoroskan Ascent (Thermo, San Jose, CA, USA) with an excitation wavelength of 485 nm and an emission wavelength of 630 nm. Readings were taken at 30 min intervals for 4 h.

*Membrane potential:* Mitochondrial membrane potential was measured by incubating control or mIMCD cells with 50 nM tetramethylrhodamine ethyl ester (TMRE; Invitrogen) for 30 min, followed by fluorescence-activated cell sorter (Coulter Elite Flow Cytometer, Beckman Colter Electronics Ltd., Hialech, FL, USA) analysis. The fluorescence intensity was monitored at 582 nm (FL-2 channel) using FACScan (15,000 cells/sample).

### 4.12. Immunohistochemistry

Immunohistochemistry was performed on 4 µm sections of each paraffin block. The sections were deparaffinized and rehydrated and were then subjected to antigen retrieval performed according to several recommended methods. Endogenous peroxidase activity was blocked using 0.3% hydrogen peroxide. The sections were incubated with primary antibodies overnight at 4 °C. The primary antibodies used were as follows: F4/80 (sc-59171, monoclonal, rat, Santa Cruz Biotechnology), α-SMA (ab5694, polyclonal, rabbit, Abcam, Cambridge, MA, USA), MCP-1(ab7202, polyclonal, rabbit, Abcam, Cambridge, MA, USA), OPN (ab8448, polyclonal, rabbit, Abcam, Cambridge, MA, USA), TGF-β (MAB 1032, monoclonal, mouse, Chemicon, Temecula, CA, USA), and 8-OHdG (MOG-020P, mouse, monoclonal, Japan Institute for the Control of Aging, Shizuoka, Japan). After washing, the secondary antibody was applied as follows: for F4/80; LS-J1022-1 (LSbio, Seattle, WA, USA), for α-SMA, MCP-1, and OPN; P0448 (Agilent DAKO, Santa Clara, CA, USA), and for TGF8- β and OHdG; P0447 (Agilent DAKO, Santa Clara, CA, USA). The samples were further incubated for 30 min at room temperature. Finally, the slides were visualized by DAB immunostaining using the REAL EnVision Detection System, Peroxidase/DAB+ (Agilent DAKO, Santa Clara, CA, USA).

### 4.13. Statistical Analysis

The data are reported as the mean ± standard deviation. All statistical analyses were performed using Prism 9 (GraphPad Software, Inc., San Diego, CA, USA). Multiple comparisons among groups were performed using a Kruskal–Wallis non-parametric ANOVA test with Dunn’s test for multiple comparisons. An unpaired Student’s t-test was used to compare baseline parameters between WT and CRIF1 KO mice. The difference between groups was considered significant when the *p*-value was less than 0.05.

## Figures and Tables

**Table 1:** Baseline parameters in wild-type and CR6-interacting factor-1 knockout mice.

	WT	CRIF1-KO	*p* Value
Arterial blood			
pH	7.22 (±0.06)	7.18 (±0.01)	ns
Pco2(mmHg)	47.80 (±11.48)	54.33 (±1.53)	ns
HCO_3_-(mEq/L)	19.26 (2.29)	20.47 (±0.83)	ns
Serum			
Urea Nitrogen (mg/dL)	12.10 (±1.29)	10.90 (±1.43)	ns
Creatinine (mg/dL)	0.04 (±0.02)	0.03 (±0.03)	ns
Na (mEq/L)	142.8 (±2.37)	150.6(4±8.82)	ns
K (mEq/L)	3.6 (±0.31)	3.9 (±0.63)	ns
Cl (mEq/L)	93.1 (±2.04)	98 (±6.34)	ns
Urine			
24 h Urine Volume(ml/g.bwt)	0.059 (± 0.021)	0.065 (±0.017)	ns
Urea Nitrogen (mg/dL)	522.44 (±207.0)	298.04 (±173.69)	ns
Creatinine (mg/dL)	9.65 (±40.77)	6.10 (±3.24)	ns
Na (mEq/L)	32.04 (±7.51)	21.82 (±11.95)	ns
K (mEq/L)	35.68 (±14.87)	17.34 (±12.42)	ns
Cl (mEq/L)	32.52 (±10.11)	18.82 (±15.53)	ns
ACR	60.1 (±30.08)	81.3 (±16.53)	ns

## Supplementary Materials

The following are available online at https://www.mdpi.com/article/10.3390/ijms222111699/s1.

## Abbreviations

UUO	Unilateral Ureteral Obstruction
ATP	Adenosine triphosphate
CRIF1	CR6-interacting factor-1
OCR	oxygen consumption rate
CCCP	chain decoupler carbonyl cyanide m-chlorophenylhydrazone
mIMCD	inner medullary collecting duct
MCP-1	Monocyte Chemoattractant Protein-1
OPN	osteopontin
CKD	chronic kidney disease

## References

[1] Parrish A.R. (2016). Advances in chronic kidney disease. Int. J. Mol. Sci..

[2] Chevalier R.L. (2016). The proximal tubule is the primary target of injury and progression of kidney disease: Role of the glomeru-lotubular junction. Am. J. Physiol.-Renal.

[3] Fujiu K., Manabe I., Nagai R. (2011). Renal collecting duct epithelial cells regulate inflammation in tubulointerstitial damage in mice. J. Clin. Investig..

[4] Choi A., Nam S.A., Kim W.-Y., Park S.H., Kim H., Yang C.W., Kim J., Kim Y.K. (2018). Notch signaling in the collecting duct regulates renal tubulointerstitial fibrosis induced by unilateral ureteral obstruction in mice. Korean J. Intern. Med..

[5] Poyton R.O., Ball K., Castello P.R. (2009). Mitochondrial generation of free radicals and hypoxic signaling. Trends Endocrinol. Metab..

[6] Kim J., Jang H.-S., Park K.M. (2010). Reactive oxygen species generated by renal ischemia and reperfusion trigger protection against subsequent renal ischemia and reperfusion injury in mice. Am. J. Physiol. Renal Physiol..

[7] Chacko B., Reily C., Srivastava A., Johnson M.S., Ulasova E., Agarwal A., Zinn K., Murphy M.P., Kalyanaraman B., Darley-Usmar V. (2010). Prevention of diabetic nephropathy in Ins2^+/-AkitaJ^ mice by the mitochondria-targeted therapy Mito Q. Biochem. J..

[8] Santos N.A., Catao C.S., Martins N.M., Curti C., Bianchi M.L., Santos A.C. (2007). Cisplatin-induced nephrotoxicity is asso-ciated with oxidative stress, redox state unbalance, impairment of energetic metabolism and apoptosis in rat kidney mito-chondria. Arch. Toxicol..

[9] Schwerdt G., Freudinger R., Schuster C., Weber F., Thews O., Gekle M. (2005). Cisplatin-induced apoptosis is enhanced by hypoxia and by inhibition of mitochondria in renal collecting duct cells. Toxicol. Sci..

[10] Wang J., Biju M.P., Wang M.-H., Haase V.H., Dong Z. (2006). Cytoprotective Effects of hypoxia against cisplatin-induced tubular cell apoptosis: Involvement of mitochondrial inhibition and p53 suppression. J. Am. Soc. Nephrol..

[11] Chung H.K., Yi Y.W., Jung N.-C., Kim D., Suh J.M., Kim H., Park K.C., Song J.H., Kim D.W., Hwang E.S. (2003). CR6-interacting factor 1 interacts with gadd45 family proteins and modulates the cell cycle. J. Biol. Chem..

[12] Nakayama K., Nakayama N., Wang T.-L., Shih I.-M. (2007). NAC-1 Controls cell growth and survival by repressing transcription of gadd45gip1, a candidate tumor suppressor. Cancer Res..

[13] Na K., Jeong J., Shin J., Chang Y.-K., Suh K.-S., Lee K., Choi D. (2021). Mitochondrial dysfunction in podocytes caused by crif1 deficiency leads to progressive albuminuria and glomerular sclerosis in mice. Int. J. Mol. Sci..

[14] Roy A., Al-Bataineh M.M., Pastor-Soler N.M. (2015). Collecting Duct intercalated cell function and regulation. Clin. J. Am. Soc. Nephrol..

[15] Lote C.J., Harper L., O Savage C. (1996). Mechanisms of acute renal failure. Br. J. Anaesth..

[16] Klahr S., Morrissey J. (2002). Obstructive nephropathy and renal fibrosis. Am. J. Physiol. Renal Physiol..

[17] Kinter M., Wolstenholme J.T., Thornhill B.A., Newton E.A., McCormick M.L., Chevalier R.L. (1999). Unilateral ureteral ob-struction impairs renal antioxidant enzyme activation during sodium depletion. Kidney Int..

[18] Xie P., Sun L., Nayak B., Haruna Y., Liu F.Y., Kashihara N., Kanwar Y.S. (2009). C/EBP-beta modulates transcription of tubu-lointerstitial nephritis antigen in obstructive uropathy. J. Am. Soc. Nephrol..

[19] Brownlee M. (2001). Biochemistry and molecular cell biology of diabetic complications. Nat. Cell Biol..

[20] Brownlee M. (2005). The Pathobiology of Diabetic Complications: A Unifying Mechanism. Diabetes.

[21] Vaziri N.D., Dicus M., Ho N.D., Boroujerdi-Rad L., Sindhu R.K. (2003). Oxidative stress and dysregulation of superoxide dis-mutase and NADPH oxidase in renal insufficiency. Kidney Int..

[22] Paravicini T.M., Touyz R.M. (2008). NADPH oxidases, reactive oxygen species, and hypertension: Clinical implications and ther-apeutic possibilities. Diabetes Care.

[23] Satoh M., Fujimoto S., Haruna Y., Arakawa S., Horike H., Komai N., Sasaki T., Tsujioka K., Makino H., Kashihara N. (2005). NAD(P)H oxidase and uncoupled nitric oxide synthase are major sources of glomerular superoxide in rats with experimental diabetic nephropathy. Am. J. Physiol. Physiol..

[24] Murphy M.P. (2008). How mitochondria produce reactive oxygen species. Biochem. J..

[25] Skulachev V.P. (2013). Cationic antioxidants as a powerful tool against mitochondrial oxidative stress. Biochem. Biophys. Res. Commun..

[26] Marchi S., Giorgi C., Suski J.M., Agnoletto C., Bononi A., Bonora M., De Marchi E., Missiroli S., Patergnani S., Poletti F. (2011). Mitochondria-ros crosstalk in the control of cell death and aging. J. Signal Transduct..

[27] Ishimoto Y., Tanaka T., Yoshida Y., Inagi R. (2018). Physiological and pathophysiological role of reactive oxygen species and reactive nitrogen species in the kidney. Clin. Exp. Pharmacol. Physiol..

