# Supplementary material for: Collecting Duct-Specific CR6-Interacting Factor-1-Deletion Aggravates Renal Inflammation and Fibrosis Induced by Unilateral Ureteral Obstruction
Source: Int J Mol Sci. 2021 Oct 28;22(21):11699. doi: 10.3390/ijms222111699 (PMC8584192; doi:10.3390/ijms222111699)
Supplement: Supplementary file 1 [file ijms-22-11699-s001.zip › ijms-1421842-Supplementary.pdf]

A

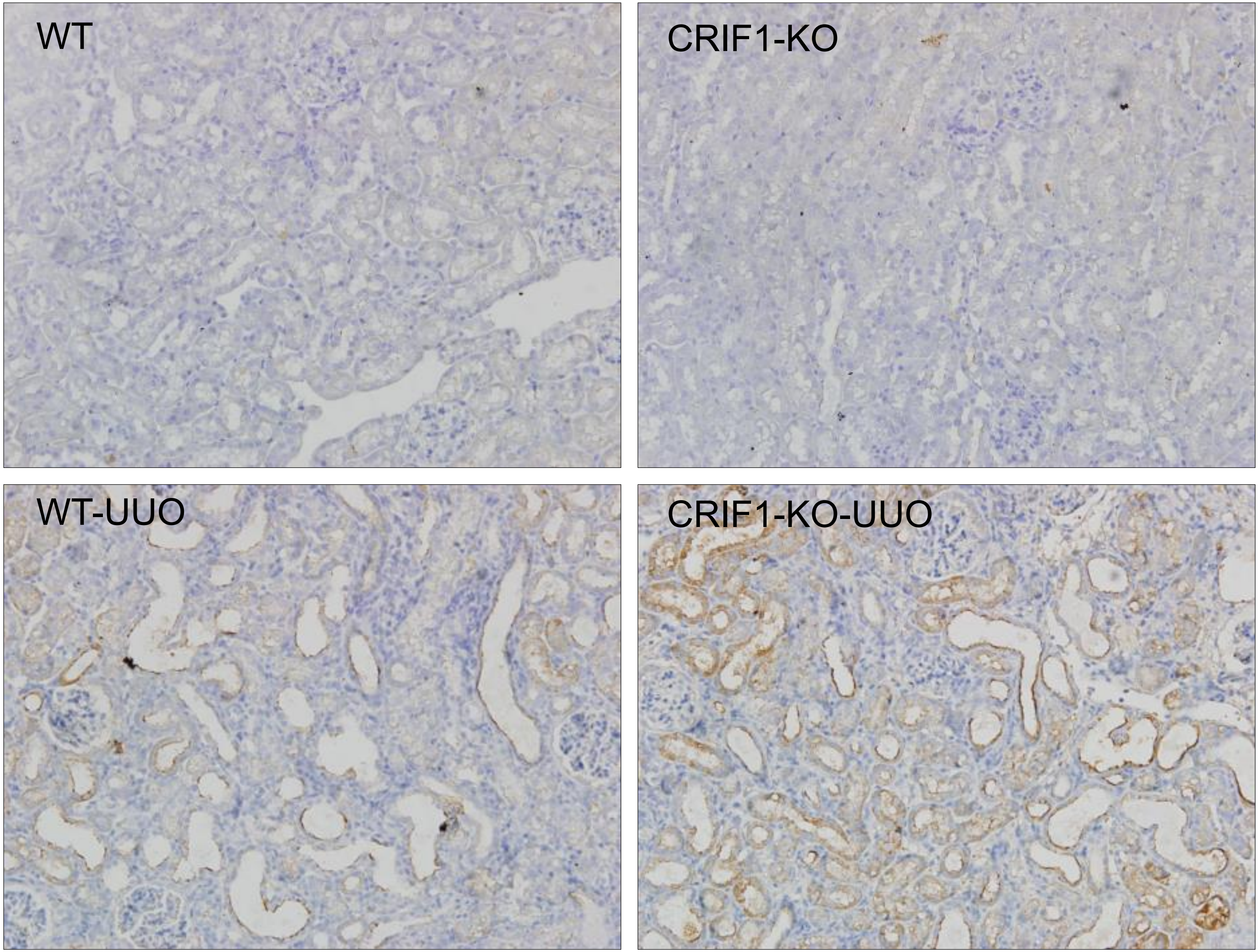

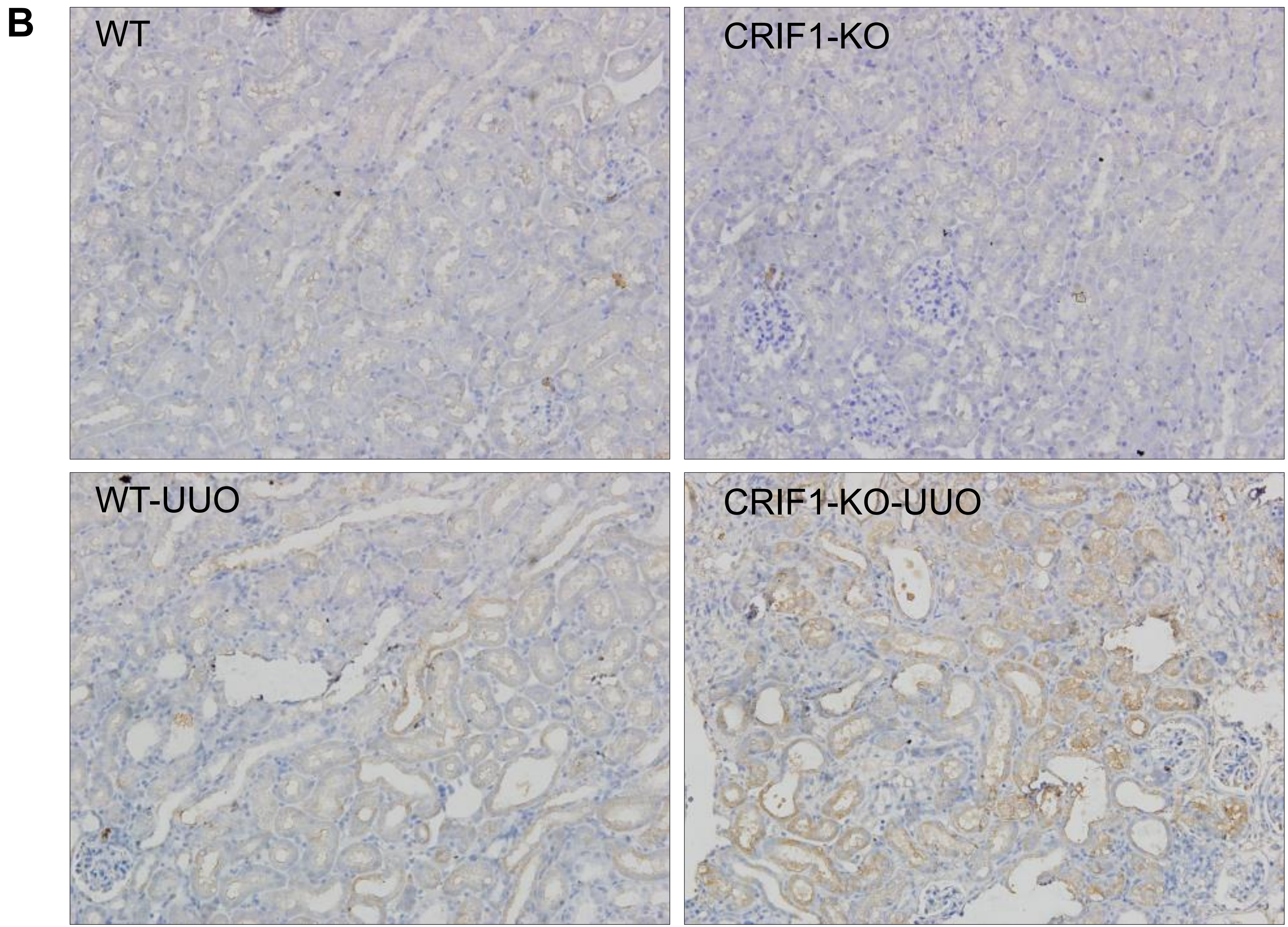

Figure S1. Magnitude images of immunohistochemistry of MCP-1 (**A**) and Osteopontin (**B**), WT.

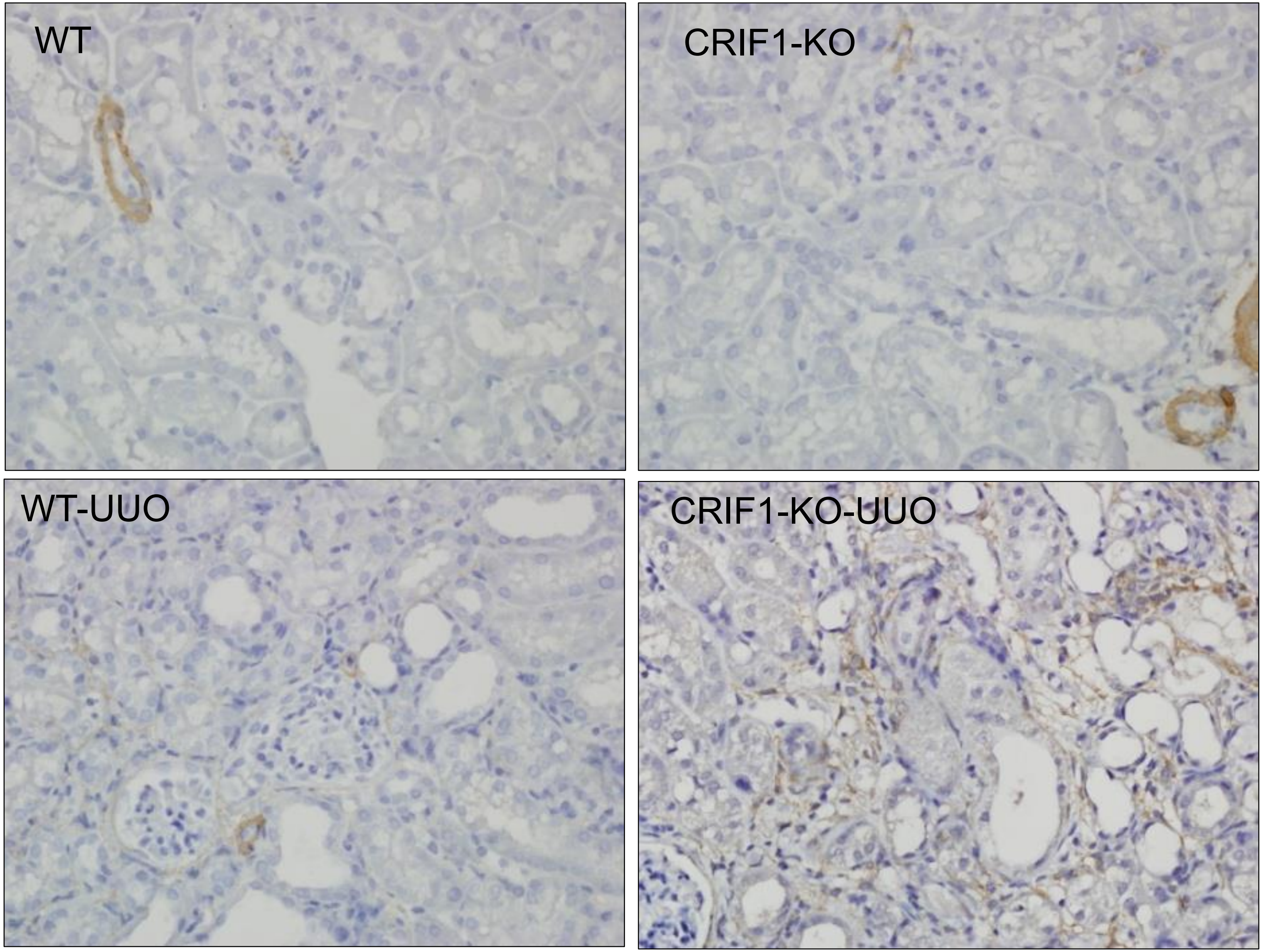

Figure S2. Magnitude images of immunohistochemistry of a-SMA. WT.
